# The Clinical Efficacy and Safety of the Sahastara Remedy versus Diclofenac in the Treatment of Osteoarthritis of the Knee: A Double-Blind, Randomized, and Controlled Trial

**DOI:** 10.1155/2015/103046

**Published:** 2015-02-15

**Authors:** Piya Pinsornsak, Puritat Kanokkangsadal, Arunporn Itharat

**Affiliations:** ^1^Department of Orthopedics, Faculty of Medicine, Thammasat University, Pathumthani 12120, Thailand; ^2^Department of Applied Thai Traditional Medicine, Faculty of Medicine, Thammasat University, Pathumthani 12120, Thailand; ^3^Center of Excellence on Applied Thai Traditional Medicine Research (CEATMR), Thammasat University, Pathumthani 12120, Thailand

## Abstract

*Introduction*. The Sahastara (SHT) remedy is a Thai traditional medicine that has been acknowledged in the Thai National List of Essential Medicine and has been used as an alternative medicine to treat knee osteoarthritis. Although SHT remedies have been used in Thai traditional medical practices for a long period of time, there are few reports on their clinical trials. *Aim of the Study*. To investigate the clinical efficacy and safety of the SHT remedy in treating OA of the knee when compared to diclofenac. *Methods*. A phase 2, double-blind, randomized, and controlled trial study with a purpose to determine the clinical efficacy and safety of SHT in comparison with diclofenac for the treatment of knee osteoarthritis. Sixty-six patients, ages between 45 and 80 years of age, were randomly allocated into 2 groups. The SHT group received 1,000 mg of SHT powdered capsules 3 times per day, orally before meals, while another group received 25 mg of diclofenac sodium capsules 3 times a day, orally after meals for 28 days. All patients were followed up at 14 and 28 days for the evaluation of the efficacy and safety by using clinical examinations, blood tests, a visual analogue scale (VAS) for pain, and the 100-meter walktime test. Improvement on the quality of life was also assessed by the WOMAC index. *Results*. There were 31 and 30 patients in SHT and diclofenac groups, respectively, who had completed the study. Both medications have shown to significantly reduce the VAS for pain, and significantly improve the 100-meter walktime test and the WOMAC index score. However, there were no differences in the efficacy between the two groups. The blood chemistry showed no toxicity on renal and/or liver functions after taking SHT for 28 days but the patients who took diclofenac showed significant increases in their AST, ALT, and ALP. Systolic and diastolic blood pressure slightly increased in the diclofenac group but the SHT group did not effect on blood pressure. *Conclusions*. The SHT remedy is similar to diclofenac in all evaluating symptoms of OA knee. However, the SHT remedy has shown to be a good alternative treatment for OA knee with less systemic side effects when it was compared with diclofenac.

## 1. Introduction

Nowadays, the world as we know it has been progressing and aging, rapidly becoming a society of elderly people. The number of people 65 years of age or older was projected to increase from an estimated of 524 million in 2010 to 1.5 billion in 2050 [1]. Osteoarthritis of the knee (OA knee) is one of the most common degenerative joint diseases and is rapidly becoming a global, public health problem. OA knee leads to disability of joint function and the need for continuous treatment. The process of OA changes affect not only the articular cartilage but also the entire joint and the surrounding soft tissue. Osteoarthritis is a result of both mechanical and biological events that destabilize the normal coupling of degradation and synthesis of the articular cartilage chondrocytes, extracellular matrix, and subchondral bone. The clinical features of OA are characterized by joint pain, tenderness, limitation of movement, crepitus, the occasional effusion, and variable degrees of inflammation without systemic effects [2]. Treatments for knee osteoarthritis have been directed mostly toward the alleviation of pain and inflammation [3]. There are many therapeutic modalities for OA knee. Some of these include nonpharmacologic treatment (patient education, weight control, and physical therapy) and pharmacologic treatment (acetaminophen, nonsteroidal anti-inflammatory drugs, glucosamine with chondroitin sulphate, steroids, viscosupplement injections, and surgery). Regarding pharmacologic treatment, several countries use NSAIDs as a first line drug for treatment of OA in the knees [4]. NSAIDs inhibit cyclooxygenase enzyme (COX) and prostaglandins resulting in many side effects. Some of these side effects manifest as peptic ulcers, liver dysfunction, and renal dysfunction. The alternative medicines which have the potential for anti-inflammation with less systemic side effects may have a role in the treatment of OA of the knee.

Thai traditional medicine (TTM) is the use of alternative treatments for knee osteoarthritis which include Thai traditional massage, herbal hot compression, topical herbal medicines, and oral herbal medicines. The Sahastara (SHT) remedy has long been used as anti-inflammatory drug to relieve muscle and joint pain including the pain of OA of the knee in hospital of Thailand for more fifty years [5]. Thus, the SHT has been chosen and published in The Thai National List of Essential Medicine (NLEM) since 2011 [6]. The SHT remedy contains 21 medicinal plants with the main ingredient being pepper (*Piper Nigrum* Linn.) (Table 1).

As taken from an* in vitro* study, SHT has shown anti-inflammatory activity, which inhibits nitric oxide (NO) release and COX2 activity (IC_50_ = 2.81 and 16.97 *μ*g/mL, respectively) [7]. Moreover, piperine, which is the main ingredient of SHT remedy that is derived from peppers and long peppers, inhibited the production of PGE2 and NO induced by IL-1*β*. Piperine significantly decreases the IL-1*β*-stimulated gene expression and production of MMP-3, MMP-13, iNOS, and COX-2 in human OA chondrocytes [8]. However, there is no the scientific research regarding study the efficacy and safety of SHT in humans for OA knee and comparative with NSAID. These data should be supported using SHT in hospital of Thailand and be based for continuous study in clinical trial phase 3. From these reasons, the SHT remedy might be an alternative choice for OA knee treatment. Thus, the purpose of this study was to investigate the clinical efficacy and safety of SHT when compared with the standard NSAID, diclofenac, for the treatment of knee osteoarthritis.

## 2. Methods

### 2.1. Research Design

This study was a randomized, double-blind, and controlled trial (phase 2) designed to study the clinical efficacy and safety of the Sahastara remedy in comparison to diclofenac for the treatment of knee osteoarthritis at Thammasat University Hospital, Pathumthani Province, Thailand. This study was approved by the Medical Ethics Committee of the Faculty of Medicine, Thammasat University who accepted from FDA from Thai Government (registry number MTU-EC-TM-6-093/55).

### 2.2. Subjects

The sixty-six outpatients from Department of Orthopedics, Thammasat University Hospital, between 45 and 80 years of age, who were diagnosed with primary osteoarthritis of the knee as based on the American College of Rheumatology's clinical and radiological criteria, with no knee arthroplasty plan in 3 months and minimum pain symptom severity was ensured by visual analogue scale (VAS) score at least 20 mm. from 100 mm., were included in this study [2]. The patients rated with severe knee osteoarthritis ((grade 4) as based on the Kellgren and Lawrence radiographic system), patients with serious medical conditions such as uncontrolled hypertension (BP > 140/90 mm.Hg.), severe GI disease, congestive heart disease, and liver and renal dysfunction, and obesity patients that was assessed by body mass index (BMI), more than 32 kg/m^2^, were excluded from this study.

### 2.3. Drug Preparation

The Sahastara (SHT) remedy was prepared according to 2011 NLEM [6]. Medicinal plants were collected from many parts of Thailand or imported from other countries (Table 1). They were ground and sieved to a particular size (number 80). The powder was mixed in the formulation as shown in Table 1. The SHT powder passed standard quality controls of Thai Herbal Pharmacopeia, a contamination testing, loss on drying (moisture content), total ash content, heavy metal content, and stability in accelerated conditions testing [9]. The high performance chromatography (HPLC) was also performed to ensure composition of piperine as a main component in stability test, and piperine content in SHT was not less than 0.798 mg/g of powder drug or 19 mg/g of extract and % yield of extract as 4.2% w/w. Moreover, the SHT remedy has biological activity control such as anti-inflammatory testing by the NO inhibition testing (Griess reagent assay). The IC_50_ in the NO inhibition testing was not more than 30 *μ*g/mL [10]. All the ingredients were encapsulated in 500 mg capsules. Diclofenac sodium, 25 mg enteric-coated tablets (Voltaren), is composed of benzene-acetic acid derivative for oral administration with the mechanisms of anti-inflammatory, analgesic, and antipyretic actions. Voltaren was manufactured and distributed by Novartis Pharmaceutical Corp. (Thailand) and encapsulate in the same preparation of a 500 mg capsule. Lactose monohydrate as a placebo was prepared in a 500 mg capsule, and omeprazole (20 mg) (Miracid, Berlin) was used as an open labeled medication.

### 2.4. Procedures

Informed consent was obtained from the patients who were eligible for the study. The patients were divided randomly into 2 groups of treatment, using a computer generated program by individual who did not contact all investigators involved in trial. The patients received randomized number sequentially from secret random list. Treatment assignment was also concealed to all investigator involved in trial. Each of patients received same appearance of treatment that contains treatment code, which was opened only in medical emergency condition. The masking was successfully achieved until open after data analysis.

In trial, demographic data, clinical signs and symptoms, laboratory tests (complete blood count, fasting blood sugar, lipid profile, liver functions test, renal functions test, and urine analysis), Visual Analogue Scale (VAS) for pain, 100-meter walk times, and the Western Ontario and McMaster Universities (WOMAC) index scores were collected on first visit for baseline data and after receiving treatment on day 14 and on day 28.

### 2.5. Drug Administration

The eligible patients were divided randomly into 2 groups of treatment. The patients in group 1 received the SHT remedy at 3,000 mg/day (2 capsules of SHT three times a day before meals and 1 capsule of a placebo three times daily after meals). This is minimum dose of SHT remedy that was indicated in NLEM. The patients in group 2 received diclofenac sodium at 75 mg/day (2 capsules of placebo three times daily before meals and 1 capsule with 25 mg diclofenac sodium three times a day after meals). In addition, 20 mg of omeprazole was given twice daily to both groups for the prevention of adverse GI effects.

### 2.6. Assessment

The treatment period was completed in 28 days with the clinical and laboratory investigation follow-up assessments at the 14th and 28th days. The global assessment was executed by the patients themselves at the last visit.

The clinical efficacy for the overall treatment was evaluated at the last follow-up by assessing the VAS pain score, the 100-meter walk times, the WOMAC index scores (ranging from 0 to 96) at day 0, day 14, and day 28, and the global assessment on a 0–4 likert scale (0: none, 4: excellent). The safety outcomes were evaluated by clinical examinations and laboratory investigations.

The toxicity of drug was considered for excluding patients following by guidance for industry in toxicity grading scale of USFDA such as creatinine more than 1.7 mg/dL, BUN more than 26 mg/dL AST, and ALT more than 2.5x upper limit of normal (ULN) or ALP more than 2.0x ULN.

### 2.7. Statistical Analysis

The “paired *t*-test” or Wilcoxon's test was used to analyze the changes in the mean values from baseline to days 14 and 28 for each group. The repeated measured analysis of variance (ANOVA) or Friedman's test was used to compare these mean values between the 2 groups. The global assessment between the 2 groups was evaluated by the Chi-square of Fisher's exact test.

## 3. Results

From a total of sixty-nine patients, 3 patients were excluded from the study due to abnormal liver functions tests. The remaining 66 patients were randomized into 2 groups (33 patients in each group). There were no significant differences in their baseline characteristics data (Table 2) and the radiographic disease grading (Table 3) between the 2 groups.

**Figure 1:**
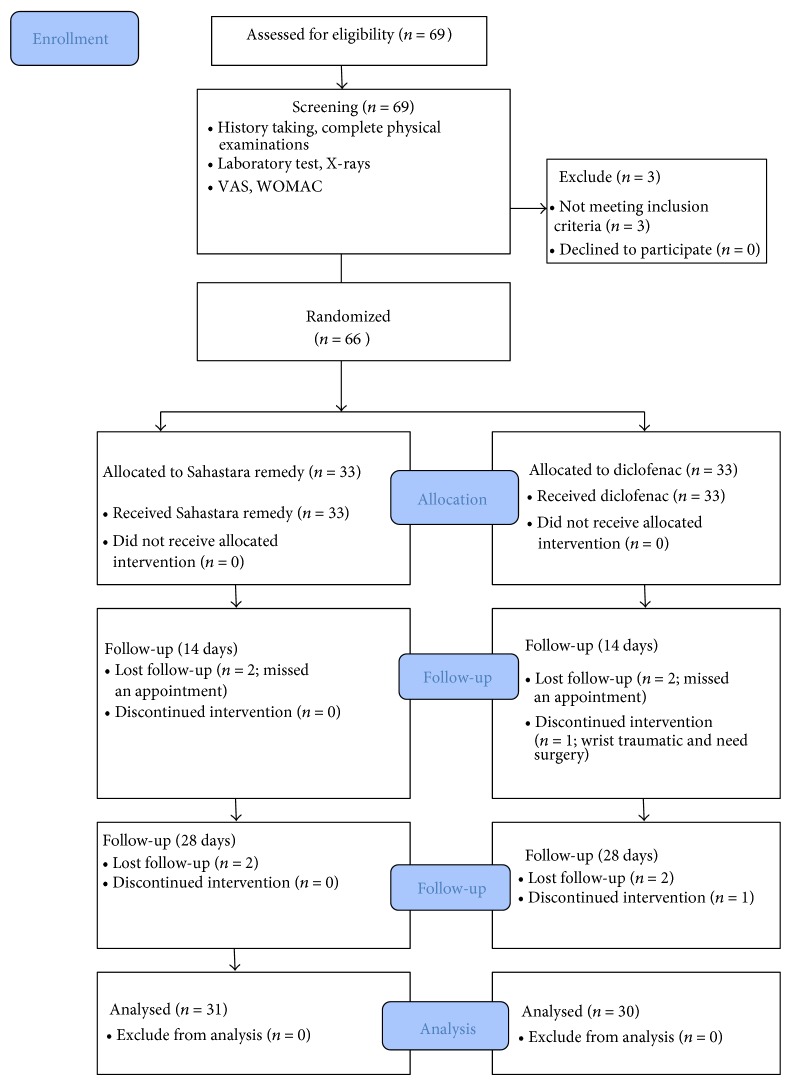
Flow of patients.

From the 66 patients, 61 (92.24%) of the patients completed the study (31 in the SHT group and 30 in the diclofenac group). Five patients dropped out at the first follow-up (4 patients who failed to follow up, and 1 patient who suffered from a traumatic wrist injury that required surgery). The results were shown in Figure 1.

### 3.1. Efficacy

At the conclusion of the study, both the SHT remedy and diclofenac had significantly reduced the VAS pain scores. The SHT remedy significantly reduced the mean VAS pain scores at days 14 and 28, while diclofenac significantly reduced the mean VAS pain scores at day 28. However, there were no significant differences between the two groups. Both groups reduced the 100-meter walk times with no statistically significant differences. The WOMAC index scores have shown decrease in both groups. Significant improvements in the physical function index and the total scores were found in the SHT group but no differences in the pain and stiffness indexes. All WOMAC index scores were significantly improved in the diclofenac group. However, there were no significant differences between the SHT and diclofenac groups in all outcomes except the stiffness index diclofenac showed better stiffness index value than SHT group significantly (Table 4).

At the end of the study, the global assessment showed improvement of symptoms in both groups but had shown no significant differences between the groups. The majority of the patients in both groups have indicated scores of “moderately better” and “very much better” (Table 5).

### 3.2. Safety

The most common adverse effect found in both groups was abdominal discomfort; it was found in SHT and diclofenac groups as 41.9% and 30%, respectively. However, there is no significant difference in both groups (Table 6). The blood pressure measurements and the systolic blood pressure in the diclofenac group at the 14th and 28th days slightly increased but SHT group did not change in either systolic or diastolic blood pressure at the 14th and 28th days. However, there is no significant difference in both groups. Liver and renal functions were tested for patients' safety. The renal function tests did not show any changes in both groups: blood urea nitrogen (BUN) and creatinine levels (Cr) when compared with the initial visit for both groups. The liver function tests and AST levels of SHT group showed significant decrease at the final visit of patients and no effect in the ALTs and ALPs for the SHT group. The AST, ALT, and ALP have shown statistically significant increases after treatment in the diclofenac group (Table 7).

## 4. Discussions

Although the SHT remedy has been used in TTM for more than 50 years, there were few reports in the literature. In the previous report, SHT showed the higher inhibitory effect on nitric oxide (NO) release as proinflammatory mediator in activated murine macrophages cell line (RAW 264.7) than indomethacin (IC_50_ = 2.81 and 20.32 *μ*g/mL, respectively) [7]. SHT preparation and its components were also tested for anti-inflammation by the inhibitory effects on LPS-stimulated PGE2 release in RAW 264.7 cells. SHT preparation and indomethacin (positive control) had IC_50_ values as 16.97 and 1.00 *μ*g/mL, respectively.* Piper nigrum* and* Piper retrofractum* which are the main plant ingredients of SHT or 33.6% of whole preparation also showed high anti-inflammatory activity on PGE2 release with IC_50_ as 17.70 and 23.08 *μ*g/mL, respectively [7]. In this previous report, SHT showed the highest inhibitory effect on NO and PGE2 when compared with its all plant ingredients, and these results were evaluated to be resulting from the synergistic effect of plant ingredients extract. However, the study of piperine, which is the main component of the SHT remedy and it is also main component of* Piper nigrum* and* Piper retrofractum, *has shown that the anti-inflammatory activity on human OA chondrocytes is by inhibiting the IL-1*β* which induces the production of PGE2 and NO [8]. These results should be concluded that SHT is anti-inflammatory effect on pain relief, reduction of inflammation, improvement of daily life activity, increases in the WOMAC scores, and decreases in the 100-meter walk times. This is a reason why SHT remedy has an equal efficacy when compared with diclofenac.

However, the SHT has many spicy plant ingredients and is recommended to be taken before meals as it can cause a burning sensation and abdominal pain which was found to be 41%. Omeprazole may not prevent the adverse GI effects from the spicy substances. More studies regarding the appropriate timing and dosage of the SHT administration need to be done. Taking SHT immediately before meals or taking more water with the medicine would help to alleviate the symptoms.

Blood pressure increase is one precaution for the use of both SHT and diclofenac. As ascertained from this study, blood pressure has shown to slightly increase in the diclofenac group but the SHT group did not show significantly change on blood pressure. However, this is the first report for the effects of the SHT remedy on blood pressure. The previous study of piperine, which is the main ingredient of the SHT remedy, has found that it decreased the percentages of iNOS, elastin, and smooth muscle cells actin SMCA and has been shown to decrease blood pressure from the third week of treatment [11]. Piperine was partially responsible for preventing the increase in blood pressure. This reason could be against the precaution of SHT remedy about increasing of blood pressure. However, this study was a short-term study, accordingly more studies regarding long-term side effect need to be done.

This study confirms the results of a previous study of 17,289 arthritis patients who took diclofenac for 18 months and showed elevation of the AST or ALT levels because diclofenac is associated with aminotransferase elevations, especially in the first 4–6 months of use [12]. However, there is no chronic toxicity report of SHT on liver function but there are the studies of acute and chronic toxicity of pepper in rats. Pepper had no liver toxicity [13]. This discovery will be suggested for OA patients who had the abdominal liver function and high level of AST and ALT. However, SHT in form of National List of Essential Medicine of Thailand used as powder drug which was not extracted and it have long been used to be anti-inflammatory condition. These results will be used in scientific data support of SHT for one choice of medical doctor used. In the future, the SHT remedy extract should be developed to modern herbal drug and design to test anti-inflammatory activity and toxicity in animal model. The molecular biology of SHT extract on anti-inflammatory effect should be also tested.

However, this study had the limitation that it was a sex recruitment because this study design did not block random for patients to be female or male. The sex depend on the out patients who visited at orthopedics department in that time. However, it was related to incidence of OA knee patients. This report showed most of patients to be female (90% in both groups). In addition, this is a short-term study because the limitation of NSAIDs use has the cautions of long-term used. However, the chronic toxicity of SHT in animal model should be continuously studied because it will be a drug of choice of chronic OA patients.

This research is the first report which did clinical trial in OA patients and compared with diclofenac. This report could be supported using SHT in hospital of Thailand.

## 5. Conclusions

The SHT remedy showed an equally clinical efficacy in alleviating symptoms of OA knee when comparing it with diclofenac. The anti-inflammatory effects of SHT have also shown an improved quality of life in the OA patient and also no toxicity on liver or renal function. It should be a drug of choice of OA of the knee patients who had abnormal liver function and hypertension because it showed no effect on blood pressure and liver function tested values. The SHT remedy has been proven to be a good alternative medicine for the treatment of knee osteoarthritis.

## Figures and Tables

**Table 1:** Medicinal plants in Sahastara remedy formulations (for 1,000 g. of powder drug).

Thai name	Scientific name	Voucher specimen	Part of use	Weight (g)	Collected from
Prik-Thai	*Piper nigrum* Linn.	SKP146161401	Fruit	240	Chanthaburi, Thailand
Jet-Ta-Mul-Plerng-Dang	*Plumbago indica* Linn.	SKP148160901	Root	224	Laos
Sa-Mhor-Thai	*Terminalia chebula* Retz.	SKP049200301	Fruit	104	Sa Kaeo, Thailand
Dee-Plee	*Piper retrofractum* Vahl.	SKP146160301	Fruit	96	Chanthaburi, Thailand
Tong-Tank	*Baliospermum montanum* Muell. A.	SKP121021301	Root	80	Kanchanaburi, Thailand
Wan-Nam	*Acorus calamus* Linn.	SKP015010301	Rhizome	88	Nonthaburi, Thailand
Has-Sa-Khun-Tade	*Kleinhovia hospita* Linn.	SKP183110801	Root	48	Kanchanaburi, Thailand
Ka-Ra-Boon	*Cinnamomum camphora* Linn.	SKP096030301	—	14	China
Dok-Chan	*Myristica fragrans* Houtt.	SKP121130601	Aril of seed	13	China
Luk-Chan	*Myristica fragrans* Houtt.	SKP121130601	Seed	12	China
Tien-Dang	*Lepidium sativum* Linn.	SKP057121901	Seed	11	India
Tien-Ta-Tuk-Ka-Tan	*Anethum graveolens* Linn.	SKP199010701	Fruit	10	India
Ma-Ha-Hing	*Ferula assafoetida* Linn.	SKP199060101	Resin	10	India
Tien-Sut-Ta-But	*Pimpinella anisum* Linn.	SKP199160101	Fruit	9	China
Tien-Khao	*Cuminum cyminum* Linn.	SKP199030301	Fruit	8	India
Jing-Jor	*Merremia vitifolia* (Burm. f.) Hallier f.	SKP054132201	Root	8	Kanchanaburi, Thailand
Tien-Dum	*Nigella sativa *Linn.	SKP160141901	Seed	7	China
Kote-Kag-Kra	*Anacyclus pyrethrum *(L.) DC.	SKP051011601	Root	6	China
Kote-Ka-Mao	*Atractylodes lancea* (Thunb) DC.	SKP051011201	Rhizome	5	China
Kote-Kan-Prao	*Picrorhiza kurroa* Benth.	SKP177161101	Root	4	India
Kote-Pung-Pla	*Terminalia chebula *Retz. (*gall*)	SKP019200301	Gall	3	India

**Table 2:** Baseline characteristics of patients.

Data	SHT remedy (*n* = 31)	Diclofenac (*n* = 30)	*P* value^*^
Female, number (%)	28 (90.3)	27 (90)	0.96^c^
Age; yrs, mean (SD)	60.38 (6.97)	58.23 (7.99)	0.27^t^
Weight; kg., mean (SD)	68.48 (10.64)	65.27 (9.81)	0.23^t^
Height; cm., mean (SD)	158.29 (6.38)	157.13 (5.90)	0.47^t^
BMI; Kg/m^2^, mean (SD)	27.27 (3.69)	26.35 (3.02)	0.29^t^
Visual analogue scale (VAS); mm., mean (SD)	44.1 (23.5)	43.5 (19.3)	0.91^t^
100-meter walking time; sec., mean (SD)	103.77 (47.51)	103.19 (35.43)	0.96^t^

WOMAC index score, mean (SD)			
Pain index	8.52 (3.77)	8.47 (2.39)	0.95^t^
Stiff index	3.32 (1.92)	3.53 (1.61)	0.65^t^
Physical function index	30.64 (11.69)	31.03 (10.77)	0.89^t^
Total score	**42.65** (**15.70**)	**43.13** (**13.69**)	0.90^t^

Laboratory data, mean (SD)			
Blood pressure			
Systolic (mm.Hg.)	123.55 (11.42)	121.83 (13.03)	0.586^t^
Diastolic (mm.Hg.)	82.90 (8.24)	80.50 (8.13)	0.256^t^
Renal function tests			
BUN (mg/dL)	13.74 (4.69)	15.35 (4.55)	0.177^t^
Creatinine (mg/dL)	0.84 (0.27)	0.80 (0.20)	0.457^t^
Liver function tests			
AST (U/L)	28 (19.49)	22.33 (10.35)	0.163^t^
ALT (U/L)	46.48 (23.94)	40.90 (12.91)	0.264^t^
ALP (U/L)	108.26 (25.95)	98.27 (23.26)	0.119^t^

^*^Statistical analysis: ^t^Student's *t*-test, ^c^chi-square test.

**Table 3:** The radiographic grading at entry into the study.

Kellgren and Lawrence X-ray grade	SHT remedy (*n* = 31)	Diclofenac (*n* = 30)	*P* value^*^
Grade 1	2	1	0.610^c^
Grade 2	17	20	
Grade 3	12	9	

^*^Statistical analysis: ^c^chi-square test.

**Table 4:** The efficacy outcome of Sahastara remedy and diclofenac.

Data^*^	Follow-up	Treatments		*P* value^**^
		SHT remedy	Diclofenac	
Visual Analogue Scale (VAS) (mm.)	Day 0	44.1 (23.5)	43.5 (19.3)	0.590^r^
	Day 14	31.8 (22.8)^††^	36.3 (24.3)	
	Day 28	27.4 (18.3)^†††^	31.5 (23.5)^††^	

100-meter time walk (seconds)	Day 0	103.77 (47.51)	103.19 (35.43)	0.097^f^
	Day 14	98.55 (25.21)	101.37 (24.39)	
	Day 28	96.58 (24.09)	95.92 (18.77)	

WOMAC index score				
Pain index	Day 0	8.52 (3.77)	8.47 (2.39)	0.545^r^
	Day 14	7.94 (3.05)	7.43 (3.92)	
	Day 28	7.06 (3.31)	6.30 (3.70)^†††^	

Stiff index	Day 0	3.32 (1.92)	3.53 (1.61)	0.007^f^
	Day 14	3.42 (1.86)	2.40 (1.79)^†††^	
	Day 28	2.90 (1.87)	2.5 (1.78)^††^	

Physical functions index	Day 0	30.64 (11.69)	31.03 (10.77)	0.836^r^
	Day 14	30.61 (12.54)	27.57 (12.26)^†^	
	Day 28	22.48 (12.17)^††^	23.50 (13.06)^†††^	

Total score	Day 0	42.65 (15.70)	43.13 (13.69)	0.643^r^
	Day 14	41.32 (15.69)	37.40 (16.55)^††^	
	Day 28	34.61 (16.21)^†^	33.00 (18.02)^†††^	

^*^Data represent mean (SD), ^**^statistic analysis: ^r^repeated measured ANOVA, ^f^Friedman's test.

^†^Significant difference from day 0 within group (*P* < 0.05), ^††^significant difference from day 0 within group (*P* ≤ 0.01), and ^†††^significant difference from day 0 within group (*P* ≤ 0.001).

**Table 5:** Overall assessment of treatment evaluated at day 28th.

Global assessment (point)	SHT (*n* = 31) Number (%)	Diclofenac (*n* = 30) Number (%)	*P* value^*^
0: none	2 (6.5)	0 (0)	0.572^c^
1: mild better	6 (19.4)	4 (13.3)	
2: moderate better	13 (41.9)	13 (43.3)	
3: very much better	9 (29.0)	11 (36.7)	
4: excellent	1 (3.2)	2 (6.7)	

^*^Statistical analysis: ^c^chi-square test.

**Table 6:** Adverse events of Sahastara remedy and diclofenac.

Adverse events	SHT (*n* = 31) Number (%)	Diclofenac (*n* = 30) Number (%)
Abdominal discomfort	13 (41.9)	9 (30)
Constipation	1 (3.22)	1 (3.33)
Dry lips and throat	1 (3.22)	1 (3.33)
Sweating	1 (3.22)	2 (6.66)
Dizziness	1 (3.22)	1 (3.33)

**Table 7:** Blood pressure, renal functions, and liver functions in safety issue.

Data^*^	Follow-up	Treatment		*P* value^**^
		SHT	Diclofenac	
Blood pressure				
Systolic blood pressure (normal ≤ 140 mm.Hg.)	Day 0	123.55 (11.42)	121.83 (13.03)	0.662^r^
	Day 14	126.77 (10.13)	127.00 (14.42)^†^	
	Day 28	123.54 (12.79)	128.67 (17.95)	

Diastolic blood pressure (normal ≤ 90 mm.Hg.)	Day 0	82.90 (8.24)	80.50 (8.13)	0.680^r^
	Day 14	84.03 (6.38)	83.50 (7.08)	
	Day 28	83.55 (7.09)	84.67 (6.81)^†^	

Renal functions				
Blood urea nitrogen; BUN (mg/dL) (ref. range = 7.0–18.0)	Day 0	13.74 (4.69)	15.35 (4.55)	0.156^r^
	Day 14	13.98 (3.87)	14.70 (4.67)	
	Day 28	13.54 (3.89)	14.96 (3.52)	

Creatinine (mg/dL) (ref. range = 0.7–1.3)	Day 0	0.84 (0.27)	0.80 (0.20)	0.603^r^
	Day 14	0.82 (0.22)	0.79 (0.23)	
	Day 28	0.81 (0.24)	0.80 (0.20)	

Liver functions				
AST (U/L) (ref. range = 15–37)	Day 0	28.00 (19.50)	22.33 (10.35)	0.452^r^
	Day 14	25.77 (12.54)	30.50 (20.88)	
	Day 28	21.61 (7.05)^ †^	29.00 (14.93)^ †^	

ALT (U/L) (ref. range = 30–65)	Day 0	46.48 (23.94)	40.90 (12.92)	0.002^f^
	Day 14	44.13 (23.95)	55.70 (36.66)^†††^	
	Day 28	41.03 (11.45)	56.37 (32.33)^†††^	

ALP (U/L) (ref. range = 50–136)	Day 0	108.26 (25.95)	98.27 (23.26)	0.198^r^
	Day 14	110.13 (46.30)	98.23 (21.85)	
	Day 28	113.84 (35.68)	106.17 (30.68)^††^	

^**^Statistical analysis: ^r^repeated measured ANOVA, ^f^Friedman's test.

^†^Significant difference from day 0 within group (*P* < 0.05), ^††^significant difference from day 0 within group (*P* ≤ 0.01), and

^†††^significant difference from day 0 within group (*P* ≤ 0.001).
